# Plasma Donors in the Southwestern United States Positively Contribute to the Diverse Therapeutic Antibody Profile of Immune Globulin Products

**DOI:** 10.1038/s41598-020-63794-y

**Published:** 2020-04-22

**Authors:** Jonathan M. Ciencewicki, Katherine R. Schouest, Todd M. Gierman, Peter J. Vandeberg, Barry D. Gooch

**Affiliations:** Grifols Bioscience Research Group; Biomat USA, Inc., Research Triangle Park, NC 27709 USA

**Keywords:** Immunology, Immunotherapy

## Abstract

Human-plasma-derived immune globulin (IG) is used in augmentation therapy to provide protective levels of antibodies to patients with primary immune deficiency diseases (PIDD) and for prophylaxis against infectious diseases. To maintain the breadth of antibodies necessary for clinical protection, it is important to understand regional patterns of antibody seroprevalence in source plasma from which IG products are manufactured. In this study, source plasma from donation centers in various locations of the Southwestern quarter of the United States was surveyed for antibody titers to hepatitis A virus (HAV), measles virus (MeV), and cytomegalovirus (CMV). A broad range of anti-HAV Ig plasma titers was observed among these centers, with some centers exhibiting 3–5 times the titers of the others. Minor to no differences were observed for levels of anti-MeV and anti-CMV, respectively. Importantly, elevated anti-HAV Ig titers were broadly observed across plasma units obtained from the centers exhibiting high titers, indicative of a potential regional phenomenon among donors as opposed to few donors with singularly high titers. Plasma from these high-titer centers conferred significantly greater neutralization against HAV *in vitro*. The outcomes of this study give a glimpse of the antibody diversity inherent in human plasma used to manufacture IG products..

## Introduction

Immune globulin (IG) products made from human plasma are used in augmentation therapy to provide antibodies critical for the protection of persons with primary immune deficiency diseases (PIDD), as well as for prophylaxis of various infectious diseases. The broadly-protective properties of the antibodies in IG products stem from their human origin and because they are purified from pools made up of large numbers of plasma donations. IG products manufactured in the United States (US), as well as some IG products manufactured in other countries, are derived from plasma collected exclusively from donation centers in the US, which can span the entire geography of the country. Safety measures implemented nearly two decades ago—collaborative, industry-wide epidemiological surveillance of the donor population, judicious selection of donors, rigorous testing of plasma by serological and molecular (e.g., nucleic acid testing) methods and validation of manufacturing methods for virus clearance capacity—provide a high degree of confidence that IG products, regardless of plasma origin, safely deliver a diverse, natural combination of human polyclonal antibodies^1,2^.

Seroprevalence of antibodies to infectious diseases in the human population is influenced by a number of factors, including management of food and water resources, sanitation, vaccination, climate, sociodemographics, and the emergence of infectious agents, previously unknown or familiar^3^. Since the status of factors such as these can vary over time and across geographic regions, the profiles of antibodies among donors whose plasma is used to manufacture IG products are dynamic. For example, changes in viral antigen exposure in recent years have had a real impact on IG products’ antibody levels against a number of viruses, among which are hepatitis A virus (HAV) and measles virus (MeV).

In the case of HAV, declining overall rates of hepatitis A infection, due particularly to increased vaccine coverage beginning in the mid-1990s, have had the effect of reducing naturally-acquired anti-HAV titers among adult plasma donors^4–9^. Consequently, available IG preparations have been found to fall short of potency expectations^10^. This development has prompted Grifols, manufacturer of the lone IG product (GamaSTAN®) approved in the US for HAV prophylaxis, to update dosage regimens to ensure that adequate circulating anti-HAV immunoglobulin levels are reached in patients^11,12^. The use of widespread measles vaccination has had a similar effect on MeV-neutralizing antibodies in IG products for intravenous administration^13^. Nevertheless, human plasma IG augmentation therapy still offers the unique advantage of a diverse, polyvalent therapeutic antibody repertoire that cannot be obtained by any means other than purification of IG from large numbers of plasma donations collected across a wide geographic spectrum.

A better understanding of the antibody diversity with respect to geography could help to mitigate the decline in antimicrobial potencies observed in IG preparations. For example, it is known that anti-HAV seroprevalence in the US is greatest among populations residing in the Southwestern US^14,15^, a phenomenon not necessarily due to expanded immunization coverage, but more likely indicative of more potent, longer-lived naturally-acquired immunity. The present study was designed primarily to examine the anti-HAV content, and potential benefit, of plasma obtained from donors in the Southwestern quarter of the US. In addition, since it is possible this plasma possesses elevated antibody levels to other infectious agents^16^, we examined levels of antibodies to MeV and human herpesvirus 5, also known as cytomegalovirus (CMV).

## Materials and Methods

### Human plasma

Plasma was obtained from donation collection centers located in El Paso, Texas (TX); McAllen, Texas (TX); San Diego, California (CA); Midwest City, Oklahoma (OK); and Provo, Utah (UT), all of which are associated with the Southwestern US. Plasma was also obtained from one center, Clarksville, Tennessee (TN), outside of this region. The primary selection criterion for donation centers was proximity to regions of high HAV incidence and seroprevalence. While Texas and California have some of the highest incidence and seroprevalence rates in the US, Oklahoma and Utah—which are loosely associated with the Southwest—and Tennessee have relatively low incidence rates. Texas and California also have a higher foreign born population than the other states, which may contribute to the higher seroprevalence. Beyond the selection of states, criteria were: (1) centers that perform testing at the facility to allow for quick access to samples; (2) centers that have sufficient unique donors over a short time-span to produce an adequate sample size. Donors are allowed to donate two times per week, and since unique donors were desired, it was prudent to limit the collection period of samples to a few days in order to avoid duplicate donors.

All plasma samples used in this study were residuals of samples processed as part of Grifols’ standard routine for the qualification of donors and material for further manufacture. Each donor provides informed consent that allows Grifols to conduct research on samples collected at its plasma collection centers. The informed consent documentation has been reviewed by institutional review boards (IRBs) as part of several prospective clinical studies.

All plasma units from which samples for this study were pulled were non-reactive for all serological and nucleic acid tests performed, including HAV ribonucleic acid (RNA) by real-time quantitative reverse transcription (qRT) polymerase chain reaction (PCR) testing. For the purpose of this study, to allow for higher throughput antibody testing, plasma pools were created by combining aliquots of 16 individual plasma donations from a given center per pool. The pools were stored at ≤ −20 °C until use. A total of 171 pools were created: 32 pools each from El Paso-TX, McAllen-TX, and San Diego-CA, and 25 pools each from Midwest City-OK, Provo-UT, and Clarksville-TN.

Twelve of the plasma pools were identified for further analysis of their respective individual donations: three pools each from the El Paso-TX, McAllen-TX, Midwest City-OK, and Clarksville-TN, centers. Samples were obtained from the 192 individual, unique donations that comprised the twelve pools.

### Cell culture

African green monkey kidney (BS-C-1), fetal rhesus monkey kidney (FRhK), human fetal lung fibroblast (MRC-5), and human retinal pigmented epithelial (ARPE-19) cells were obtained from ATCC (Manassas, VA) and cultured weekly in Dulbecco’s modified Eagle’s medium (DMEM; Life Technologies Corporation, Grand Island, NY) with 10% fetal bovine serum (FBS; Access Cell Culture, Vista, CA) along with non-essential amino acids and anti-biotic/mycotic additives and maintained at 37 °C and 5% CO_2_.

### Virus propagation

B-SC-1 cells (seeded 24 h prior) were infected with HAV (HM175/18 f; ATCC) at a multiplicity of infection (MOI) of 0.05 TCID_50_ per cell. After incubation at 35 °C for 7 d, a cell lysate was prepared and stored frozen at −70 °C. Thawed lysate was treated with Trixton-X 100 and lithium dodecyl sulfate and concentrated via tangential flow filtration (TFF). The TFF retentate was centrifuged at approximately 85000 × g for 1.5 h at 5 °C in order to force the virus into a pellet, after which the pellet was suspended in buffer and the buffer was exchanged by ultrafiltration. The virus preparation was stored at −70 °C until use.

A strain of CMV (AD-169; ATCC) frequently used in laboratories has been passaged extensively in MRC-5 fibroblasts such that it no longer exhibits tropism for naturally permissive epithelial/endothelial cells. In order to obtain a more clinically-relevant system, endothelial ARPE-19 cells were infected with AD-169 at an MOI of 0.1-1.0 TCID_50_ per cell, and the cells were serially passaged post infection until a desirable CMV titer was achieved. Adaptation of the virus did not impact its ability to infect MRC-5 fibroblasts (data not shown). The supernatant from infected cells was used without further purification in neutralization experiments.

### Enzyme-linked immunosorbent assay

A commercially-available enzyme-linked immunosorbent assay (ELISA) (Bio-Rad MONOLISA™ Anti-HAV EIA Kit, cat#72496) was used to estimate the anti-HAV Ig (IgG and IgM) titer in a plasma sample. An in-house protocol was followed to convert the ELISA to a semi-quantitative format. With each assay plate was included a series of dilutions of the kit’s calibrator, from which a standard curve was generated. A plasma sample was diluted such that its absorbance value was within the range of the standard curve, and an IG titer was estimated from a linear fit of the standard curve. The IG titer was then corrected according to the dilution factor.

Commercially-available ELISAs (Abcam anti-CMV IgG Human ELISA Kit cat#ab108724 or Abcam anti-MeV virus IgG Human ELISA Kit cat# ab108750) were used to semi-quantitatively determine the amount of anti-CMV or anti-MeV IgG in a plasma sample. The assay was performed according to the manufacturer’s instructions.

### Virus neutralization assay

A single plasma sample, neat or diluted in Hank’s buffered salt solution (HBSS), was added to the first well of each row of a 96-well microtiter plate. An equal volume of HBSS was added to the remaining wells of the entire plate. The plasma in these first wells was serially diluted across the succeeding nine wells of a respective row. An equal volume of a virus preparation was added to the same wells such that the concentration of virus in each well was 6.0 to 6.5 TCID_50_·mL^−1^. A positive control was included on each plate by adding the virus preparation to the wells of a single column containing only HBSS. A negative control was also included in which HBSS alone was left in the wells of a single column. The plate was then incubated for 60 min at 37 °C. Following the incubation, 50 µl from each well were used to inoculate a corresponding well on a microtiter plate that had been seeded with recipient cells (FRhK for HAV and ARPE-19 for CMV; MeV were not evaluated). The recipient cells were incubated with the inoculum for 2 h at 37 °C. Following infection, the inoculum was removed, maintenance medium was added, and the cells were incubated at 35 °C for HAV or 37 °C for CMV. Twenty-one days post inoculation, the cells were microscopically examined for the visual appearance of a cytopathic effect (CPE).

The virus neutralization factor of a given plasma sample was calculated as the reciprocal of the largest dilution at which not less than 50% of its inoculated wells were negative for CPE. In this case, each dilution had eight replicates (the eight wells comprising a column of a microtiter plate); therefore, the last dilution in the series that exhibited at least four CPE-negative wells dictated the neutralization factor for the plasma sample on test.

### Statistical analyses

Statistical analyses of data were performed using SAS JMP (v14) and Graphpad Prism (v8) software. For analyses of grouped data sets, an unpaired two-tail t test was used to determine if means differed significantly. For the analysis of collection centers, a one-way analysis of variance (ANOVA) was used to determine if the means differed significantly, and Tukey’s honestly significant difference (HSD) post hoc test was used to directly compare means of each center.

## Results

### Anti-HAV, -MeV, and -CMV Immune Globulin Seroprevalence in Source Plasma from US Southwest

**Figure 1:**
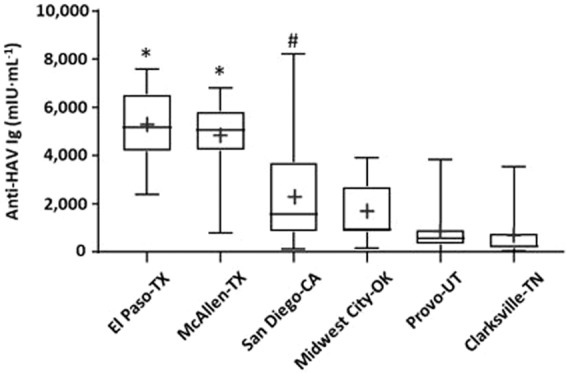
Anti-HAV Ig titers of plasma pools segregated by donor center. Plots demonstrate the spread of observed anti-HAV Ig titers measured by ELISA. The box whiskers denote the minimum and maximum titers; the bottom and top borders of a box represent the first and third quartile markers, respectively; the line within each box denotes the median, and the (+) symbol denotes the mean. (*) Mean is significantly greater than those of San Diego-CA, Midwest City-OK, Provo-UT, and Clarksville-TN, centers (p < 0.001; ANOVA, Tukey’s HSD). (#) Mean is significantly greater than those of Provo-UT, and Clarksville-TN, centers (p < 0.0001; ANOVA, Tukey’s HSD).

All 171 plasma pools were screened by an ELISA for anti-HAV Ig (IgG and IgM). When analyzed per center (Fig. 1), the mean anti-HAV Ig titers of the plasma pools from El Paso-TX (5,267 ± 1,300 mIU·mL^−1^) and McAllen-TX (4,838 ± 1,406 mIU·mL^−1^) were comparable to each other and significantly (p < 0.0001) higher than the mean titers of all other centers. The mean anti-HAV Ig titers of pools from the San Diego-CA (2,296 ± 2,052 mIU·mL^−1^) and Midwest City-OK (1,665 ± 1,131 mIU·mL^−1^) centers were comparable; the mean titer of San Diego-CA pools was significantly (p < 0.001) higher than those of Provo-UT and Clarksville-TN.

**Figure 2:**
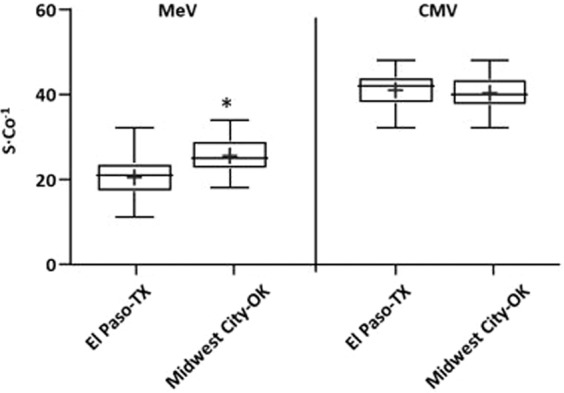
Anti-MeV and anti-CMV IgG titers of plasma pools from select donor centers. Plots demonstrate the spread of anti-MeV and anti-CMV IgG ELISA observations—reported as signal-to-cutoff (S·Co^−1^)—in plasma pools from high-titer (El Paso-TX) and mid-titer (Midwest City-OK) donor centers. The box whiskers denote the minimum and maximum titers; the bottom and top borders of a box represent the first and third quartile markers, respectively; the line within each box denotes the median, and the (+) symbol denotes the mean. (*) Mean is greater than that of the El Paso, TX, center (p < 0.0001; unpaired two-tail t test).

The 32 plasma pools from El Paso-TX and the 25 pools from Midwest City-OK were analyzed for anti-MeV and anti-CMV IgG content (Fig. 2). The level of anti-MeV IgG calculated for Midwest City-OK pools was found to be slightly higher compared to that of pools from El Paso-TX (p < 0.0001). There was no significant difference in levels of anti-CMV IgG between the two sets of pools.

### Individual unit contributions to anti-HAV IG mean pool titers

To determine if differences in pool titers were due to one or a few donors in a given pooled sample or due to an overall higher prevalence in the population, samples from the individual donations comprising twelve of the plasma pools were tested for anti-HAV Ig titers. It was necessary to choose a subset of the pools in order to maintain a feasible laboratory workload. Three pools each from the El Paso-TX, McAllen-TX, Midwest City-OK, and Clarksville-TN centers were arbitrarily selected from within the second and third quartiles of the anti-HAV Ig titers for their respective centers.

**Figure 3:**
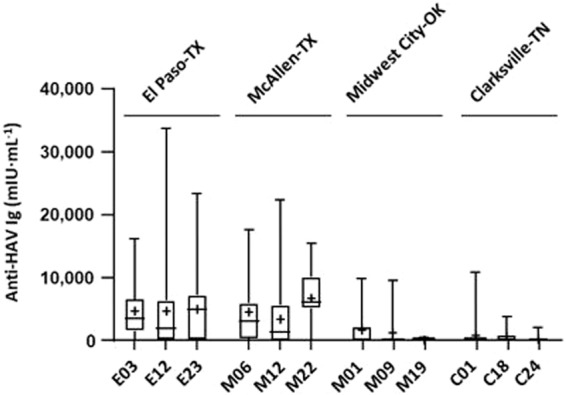
Spread of anti-HAV Ig titers of individual plasma units composing pools obtained from high-titer, mid-titer, and low-titer donor centers. Plots demonstrate the spread of anti-HAV Ig titers measured by ELISA in plasma pools from high-titer (El Paso-TX and McAllen-TX), mid-titer (Midwest City-OK), and low-titer (Clarksville, TN) donor centers. The box whiskers denote the minimum and maximum titers; the bottom and top borders of a box represent the first and third quartile markers, respectively; the line within each box denotes the median, and the (+) symbol denotes the mean.

For samples of individual donations that had composed the pools from El Paso-TX and McAllen-TX, a broad range of anti-HAV Ig titers were observed, but less than 15% of the donations exhibited values above 10,000 mIU·mL^−1^. The mean and median titers of the donations making up each pool never differed by more than approximately 2.5-fold (Fig. 3). A much lower mean anti-HAV Ig titer and a narrower range of titers were observed for the samples of individual donations pooled from centers in the other locations.

### Assessment of antiviral activity

**Figure 4:**
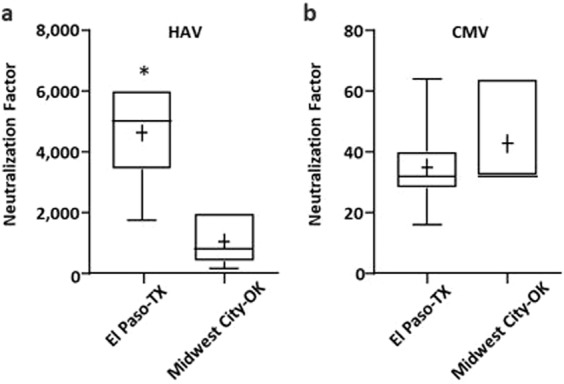
(**a**) HAV neutralization factors of select plasma pools from high-titer and mid-titer donor centers. Plots demonstrate the spread of anti-HAV activity—reported as a neutralization factor—in select plasma pools from high-titer (El Paso-TX) and mid-titer (Midwest City-OK) donor centers. The box whiskers denote the minimum and maximum factors; the bottom and top borders of a box represent the first and third quartile markers, respectively; the line within each box denotes the median, and the (+) symbol denotes the mean. (*) Mean is greater than that of the Midwest City, OK, center (p < 0.0009; unpaired two-tail t test). **(b)** CMV neutralization factors of select plasma pools from high-titer and mid-titer donor centers. Plots demonstrate the spread of anti-CMV activity—reported as a neutralization factor—in select plasma pools from high-titer (El Paso-TX) and mid-titer (Midwest City-OK) donor centers. The box whiskers denote the minimum and maximum factors; the bottom and top borders of a box represent the first and third quartile markers, respectively; the line within each box denotes the median, and the (+) symbol denotes the mean.

The same twelve pools identified for analysis of their component donations, as described in the prior section, were also evaluated for their capacity to inhibit HAV and CMV infection *in vitro* (Fig. 4a,b). Neutralization of HAV infectivity resulting from plasma pools obtained from the El Paso-TX center (mean neutralization factor of 4625) was significantly (p < 0.001) higher than what was observed for plasma pools from the Midwest City-OK center (mean neutralization factor of 1042). No significant difference was observed in the capacity to neutralize CMV. Neutralization of MeV was assayed and activity was detected, but the results were inconclusive. The method had been optimized for concentrated IG samples, not for raw plasma, and did not possess sufficient sensitivity to distinguish differences in titers between plasma samples. Further evaluation was not deemed necessary.

## Discussion

The use of pooled, plasma-derived human IG has become a critical therapy in clinical medicine^17–19^. While originally indicated as a plasma protein augmentation therapy for patients with PIDD and some secondary immunodeficiency diseases, IG has also been shown to exhibit other clinical benefits, many stemming from its anti-inflammatory and immunomodulatory effects^20,21^. It is the diverse, polyclonal nature of IG that has endowed it with its broad clinical range.

In order to maintain the therapeutic diversity of IG products, it is critical to understand patterns of antibody seroprevalence in source plasma. To this end, we tested plasma obtained from donor centers within the Southwestern quarter of the US. The data confirm that the notable anti-HAV Ig seroprevalence in particular areas of the US Southwest translates into elevated anti-HAV Ig titers in plasma collected at donation centers in those areas. Clearly, elevated antibody levels specific for HAV imply a higher incidence of infection. However, in areas where HAV is endemic, most infections occur during childhood and resolve without any lasting impact, except a robust anti-HAV response^7^. Fortunately, as suggested by the present study, healthy plasma donors emerge from such an area with elevated anti-HAV Ig titers. In fact, all plasma units from which samples for this study were pulled were negative for HAV RNA, an early marker of viral infection, by real-time qRT-PCR testing.

It is important to note that the manufacture of IG products is globally regulated and that industry practices over the past few decades have resulted in IG products with strong pathogen safety records irrespective of the geographical region within the US from which the plasma originates. Such measures include medical screening of donors, testing of plasma for disease-causing agents, and IgG purification processes that incorporate segments with validated capacities to inactivate and/or remove blood-borne pathogens, in the event they were present.

In the present study, plasma from six donation centers—five of which are in various locations of the US Southwest—were surveyed for anti-HAV Ig titers. We observed a wide range of titers among the six centers, yet three obvious groups coalesced: high-titer, mid-titer, and low-titer groups. The El Paso-TX and McAllen-TX centers yielded high-titer plasma with significantly higher anti-HAV Ig levels than those observed for all other centers surveyed. The San Diego-CA and Midwest City-OK centers produced mid-titer plasma, while the Provo-UT center and the lone non-Southwest center, Clarksville-TN, exhibited low titers.

We also examined if plasma from a high-titer center might also be enriched with other antiviral antibodies. Similar to HAV, MeV and CMV are viruses for which suitable antibody levels in IG products are clinically important^22,23^. When the full sets of plasma pools from El Paso-TX and Midwest City-OK were compared, there was no significant difference in anti-CMV IgG levels. But the anti-MeV IgG level observed in Midwest City-OK plasma was approximately 120% of that of El Paso-TX. While the result is subtle and inverted from that of anti-HAV Ig, the result is interesting nonetheless. It supports the premise that regional differences in seroprevalence helps to maintain efficacious antibody levels in IG products. Despite the recent decline in anti-MeV titers observed for some products, current IG preparations and doses are still sufficient to provide protection against MeV infection for PIDD patients^24^.

The analyses of individual units originating from select pools served to demonstrate that a number of centers in the US Southwest are capable of consistently generating plasma with elevated anti-HAV Ig titers. The elevated mean titers are not singularly driven by a few donors with extraordinarily high levels; rather, the majority of individual donations exhibit values that are not more than one standard deviation above the pool mean. A similar pattern is evident in the individual donations making up pools from centers with lower anti-HAV Ig titers. Furthermore, anti-HAV Ig titers in more than two-thirds of the individual donations from the high-titer centers were greater than the aggregate mean of plasma sample titers from the centers with lower titers. There does, however, seem to be a consistent, albeit small, number of individual donations in each high-titer pool that exhibit titers above 10,000 mIU·mL^−1^, which is most certainly an assuring contribution. In all, the high-titer centers consistently enrich plasma pools for anti-HAV Ig content.

The twelve pools described in the preceding paragraph were also used to determine how differences in anti-HAV Ig titers relate to the capacity to neutralize HAV *in vitro*. High-titer plasma pools from the El Paso-TX center demonstrated a significantly greater capacity to neutralize HAV than the plasma pools from the mid-titer Midwest City-OK center, confirming that elevated anti-HAV Ig titers associate with greater inhibition of HAV infection *in vitro*, which is consistent with previous observations^10^. More importantly, the neutralization result underscores the clinical benefit plasma from these high-titer centers provides.

This work is timely, because, despite declining overall rates of hepatitis A infection, a spike in outbreaks of late has highlighted the need for consistent IG augmentation therapy^23,25^. Moreover, MeV is reemerging as a serious public health threat with outbreaks occurring even in highly developed countries^26,27^. Notably in 2019, at the end of April the US had already seen its greatest number of reported cases since measles was said to be eliminated in 2000. Meanwhile, anti-MeV Ig titers in the general population have also been on the decline, which has caused some IG manufacturers to have had difficulty meeting the US Food and Drug Administration (FDA) product specification for anti-MeV content. To prevent shortages in product, the US FDA has lowered the specification^28^. However, as a precondition for lowering the specification, the agency recommended that firms add labeling for dosing of patients needing measles pre- or post-exposure prevention.

Herein we report the levels of anti-HAV antibodies in plasma collected from various donor centers in the Southwestern quarter of the US, and we confirmed that some locales known to harbor an elevated seroprevalence produce donors with significantly higher levels of anti-HAV antibodies (El Paso-TX and McAllen-TX) than plasma acquired from other locations loosely associated with the US Southwest (San Diego-CA, Midwest City-OK, and Provo-UT), as well as a locale outside of the region entirely (Clarksville-TN). These results are one example of how a diverse plasma supply ensures that the variety of antibodies needed for treatment of PIDD is maintained.

But a broader strategy deserves consideration. Perhaps the production of some hyperimmune IG augmentation therapies could be made more efficient and cost-effective by a rational approach that combines epidemiologic surveillance and strategic management of the plasma supply chain rather than blind screening or immunization methods. At the very least, manufacturers of IG products could attempt to understand regional differences in antibody potency stemming from natural immunity versus immunization. On a global scale, regional sourcing has the potential to be an inherent first-line defense against emerging infectious diseases in other parts of the world. Regardless of the specifics, the power of such a strategy will derive from the dynamic antibody repertoire of a diverse plasma collection platform.

## Data Availability

Data reported in this manuscript are available within the article. The datasets generated and/or analysed during the current study are available from the corresponding author upon reasonable request.
